# Human Immunodeficiency Virus Tat Protein Aids V Region Somatic Hypermutation in Human B Cells

**DOI:** 10.1128/mBio.02315-17

**Published:** 2018-04-17

**Authors:** Xiaohua Wang, Zhi Duan, Guojun Yu, Manxia Fan, Matthew D. Scharff

**Affiliations:** a Department of Cell Biology, Albert Einstein College of Medicine, Bronx, New York, USA; Columbia University Medical College

**Keywords:** AID, AIDS, B cell, HIV, Tat, somatic hypermutation

## Abstract

Long-term survivors of human immunodeficiency virus (HIV) infection have been shown to have a greatly increased incidence of B cell lymphomas. This increased lymphomagenesis suggests some link between HIV infection and the destabilization of the host B cell genome, a phenomenon also suggested by the extraordinary high frequency of mutation, insertion, and deletion in the broadly neutralizing HIV antibodies. Since HIV does not infect B cells, the molecular mechanisms of this genomic instability remain to be fully defined. Here, we demonstrate that the cell membrane-permeable HIV Tat proteins enhance activation-induced deaminase (AID)-mediated somatic hypermutation (SHM) of antibody V regions through their modulation of the endogenous polymerase II (Pol II) transcriptional process. Extremely small amounts of Tat that could come from bystander HIV-infected cells were sufficient to promote SHM. Our data suggest HIV Tat is one missing link between HIV infection and the overall B cell genomic instability in AIDS patients.

## IMPORTANCE

Although the introduction of antiretroviral therapy (ART) has successfully controlled primary effects of human immunodeficiency virus (HIV) infection, such as HIV proliferation and HIV-induced immune deficiency, it did not eliminate the increased susceptibility of HIV-infected patients to B cell lymphomas. We find that a secreted HIV protein, Tat, enhances the intrinsic antibody diversification mechanism by increasing the AID-induced somatic mutations at the heavy-chain variable (VH) regions in human B cells. This could contribute to the high rate of mutation in the variable regions of broadly neutralizing anti-HIV antibodies and the genomewide mutations leading to B cell malignancies in HIV carriers.

## OBSERVATION

Although HIV does not infect B cells, B cell abnormalities in the patients with acquired immunodeficiency syndrome (AIDS) have been observed since the beginning of the HIV outbreak (1). B cell lymphomas occur more frequently in the human HIV-infected population than in their HIV-free counterparts even in the era of antiretroviral therapy (ART) (2, 3) and represent an increasingly severe health issue for AIDS patients. Lymphomagenesis correlates with genomic instability and oncogenic mutations. Broadly neutralizing anti-HIV antibodies in long-term HIV-infected patients harbor extraordinarily high numbers of mutations, insertions, and deletions. Both phenomena raise the possibility that B cells in AIDS patients are prone to exceptional genomic instability, with the mechanisms remaining to be fully illustrated.

In B cells, activation-induced deaminase (AID) mediates the normal diversification and affinity maturation of antibodies through hypermutation of the variable (V) region genes (on-target mutagenesis) and contributes to tumorigenesis by the “off-target” mutagenesis of oncogenes and/or tumor suppressors (4). AID-mediated hypermutation is closely coupled with Pol II-mediated transcriptional events and is regulated by endogenous cellular factors that modulate transcriptional pausing and elongation (5). Since the HIV Tat protein is a well-studied transcriptional regulator that modulates gene transcriptomes in infected T cells (6) and affects bystander cells through its transmembrane capacity (7), we hypothesized that HIV Tat might affect the genomic stability of bystander B cells through its modulation of endogenous transcriptional pathways.

We chose to test our hypothesis using the human germinal center-like Ramos Burkitt’s lymphoma B cell line (8) because (i) the interaction between Tat and the polymerase II (Pol II) transcriptional machinery is not conserved in nonprimates, and (ii) primary B cells do not undergo somatic hypermutation (SHM) *ex vivo*. To efficiently study SHM at the human endogenous Ig heavy-chain V region (*Igh-V*), we established a SHM reporter system in which mCherry and the endogenous Ramos 4-34 *Igh-V* region are joined in a fusion cassette that replaces the endogenous *Igh-V* locus in Ramos B cells (9). In addition, the SHM process in this reporter cell line is mediated by a modified AID fused with the nuclear localization motif of the estrogen receptor (AID-ER fusion protein), and mutagenesis process will only occur upon tamoxifen (4-OHT) induction, which brings AID into the nucleus. SHM events on the mCherry-*Igh-V* fusion locus will lead to a loss of fluorescence that is readily quantifiable by flow cytometry.

**FIG 1:**
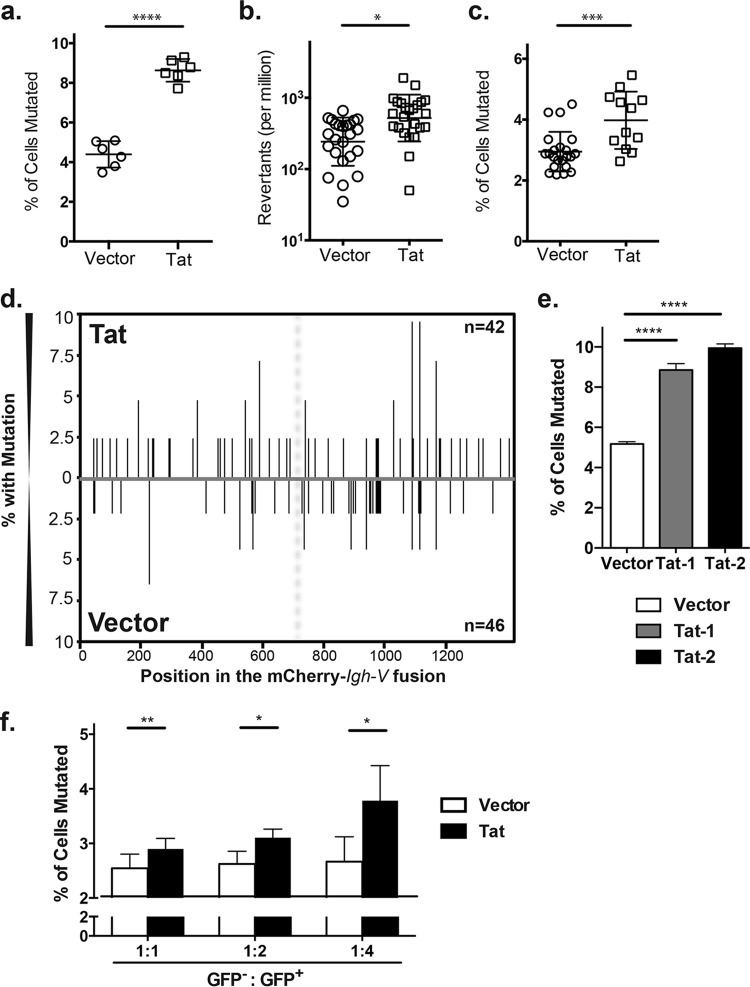
Expression of human immunodeficiency virus Tat protein promotes SHM in a human B cell line: (a) Ramos reporter cells were transduced by lentiviral particles carrying either an empty control vector or HIV-1 Tat-expressing vector. Successfully transduced cells were sorted based on GFP expression and induced by 4-OHT to transport AID into the nucleus, and the frequency of SHM was assessed 7 days later. The data represent a compiled analysis of 3 independent pairs of transductions with total of 6 independent induction experiments. (b) Ramos cells carrying a V region with a nonsense codon were transduced with either control or HIV-1 Tat-expressing constructs. Reversion frequency per million cells was analyzed using flow cytometry. Twenty-four individual clones from each experimental group were analyzed after 21 days of culture. Mutation rates were calculated using maximum likelihood. (c) Ramos reporter cells were transfected with eukaryotic expression vectors of Tat or an empty vector control, and stably transfected cell lines were selected by drug resistance. Six independent Tat-expressing clones and 9 control clones carrying the empty vector were induced to transport AID into the nucleus to assess SHM. The data represent the compiled analysis of two independent induction experiments. (d) Distribution of mutations on both strands in the reporter mCherry gene (left of the vertical dashed line) and the in-frame endogenous Ramos V region (right of the vertical dashed line) in cells transduced with either HIV-1 Tat-expressing or control vectors. The cells that had lost mCherry fluorescence were isolated by fluorescence-activated cell sorter (FACS) and then Sanger sequenced as described in Methods. The frequency of mutation at each specific site within the mCherry-*Igh-V* region fusion is shown on the *y* axis. The Sanger sequence data were analyzed by SHMTool (http://shmtool.montefiore.org) and represent the combined mutation profiles from three independent sequencing experiments. The numbers in the right-hand corners are the numbers of mCherry/VH4-34 V regions sequenced. (e) The effect of Tat-2 on SHM was determined through the mCherry reporter assay in correspondingly transduced Ramos reporter cells. Data represent 3 independent experiments. (f) Ramos cells transduced with either Tat-2-encoding or control empty vector (indicated by GFP expression) were cocultured with Ramos reporter cells not expressing Tat at the indicated ratios for 7 days after 4-OHT induction of AID. The SHM frequency of Ramos reporter cells was assessed by flow cytometry for loss of mCherry. Data represent 2 independent experiments. All data in panels e and f are shown as an average of duplicates with error bars indicating the standard deviation between the replicates. In this and Fig. 2, statistical significance is symbolized by asterisks: *, *P* < 0.05; **, *P* < 0.01; ***, *P* < 0.001; ****, *P* < 0.0001.

When the full-length 101-amino-acid (aa) Tat-1 protein was expressed in the Ramos SHM reporter cells through transduction, there was an increase in mutation reflected by approximately 2- to 2.5-fold more cells losing their fluorescence due to AID-mediated mutations than the vector control (Fig. 1a; *P* < 0.001). This observation was independently confirmed by reversion analysis in a different Ramos subclone that does not contain the mCherry cassette or inducible AID, bears an early stop codon in the endogenous wild-type heavy-chain V-coding region (10), and expresses only the endogenous AID to mediate SHM (Fig. 1b; *P* = 0.012). In Fig. 1a and b, we used lentiviruses made with a third-generation packaging system that does not contain any Tat in the packaging process. To rule out effects from other lentivirus factors, we established 12 new independent Ramos subclones stably expressing HIV Tat-1 and 12 empty vector controls from a nonlentivirus-derived eukaryotic expression vector using electroporation. With this third type of Ramos cell, we again observed that Tat-1 induced a similar statistically significant (*P* < 0.001) enhancement of SHM in the mCherry-*Igh-V* region (Fig. 1c).

When we sequenced the reporter gene cassette from the cells that had lost their mCherry fluorescence due to SHM, Tat-1 expression increased the average frequency of mutation in individual mCherry*-Igh-V* regions 1.6-fold (1.04 mutation per mCherry*-Igh-V* region in the vector control versus 1.68 mutations per mCherry-*Igh-V* region in Tat-1-expressing cells; *P* = 0.016) (Fig. 1d). When we combined this increase of mutation frequency per mutated V region with the increases in the percentage of cells that had undergone SHM revealed by the reporter and the reversion assay, HIV Tat-1 increased the overall V region mutation rate 3- to 4-fold. Similar to the wild-type cells, 50 to 60% of the G⋅C mutations in the Tat-expressing cells were in strong WRC/GYW AID hot spots. The overall distributions of mutations throughout the V region were also roughly similar in the Tat-expressing cells and the vector control cells (Fig. 1d). There are fewer mutations at A⋅T in Ramos cells than *in vivo* (11). However, 23% of the total mutations were at A⋅T sites in the Tat-expressing cells versus only 11% in the vector control cells, revealing that Tat brings the relative frequency of A⋅T mutations closer to the ~50% level seen *in vivo* (*P* = 0.016) (see Fig. S1 in the supplemental material). We further investigated whether the enhancing effect of Tat-1 on the SHM of the V region would occur with other Tat protein family members. While the Tat-2 protein from the HIV-2 virus is much less studied, and its amino acid sequence shows less than 30% identity with Tat-1 (Tat from HIV-1 virus), it shares a similar capacity of Tat-1 to interact with transcriptional factors like the P-TEFb complex (12). When Tat-2 was introduced into Ramos cells, it increased SHM to a level that was similar to if not slightly higher than Tat-1 (Fig. 1e).

10.1128/mBio.02315-17.1 FIG S1  Percentages of A/T and G/C mutations in the mCherry/VH4-34 V region in the Tat-expressing cells and the vector control. Download FIG S1, EPS file, 0.7 MB. Copyright © 2018 Wang et al. 2018 Wang et al. This content is distributed under the terms of the Creative Commons Attribution 4.0 International license.

HIV Tat is able to affect uninfected bystanders through its well-established transmembrane trafficking capacity (13, 14). To test whether paracrine Tat could promote SHM, we cocultured green fluorescent protein (GFP)-negative Ramos reporter cells either with cells containing the HIV Tat-1-coding vector (GFP positive) or with empty vector-transduced cells (also GFP positive) at the indicated ratios and found that those cultured with Tat-expressing cells mutated more than those cocultured with empty vector-transduced controls (Fig. 1f). Thus, paracrine-derived HIV Tat proteins were sufficient to promote the SHM process of the immunoglobulin V region in human B cells.

**FIG 2:**
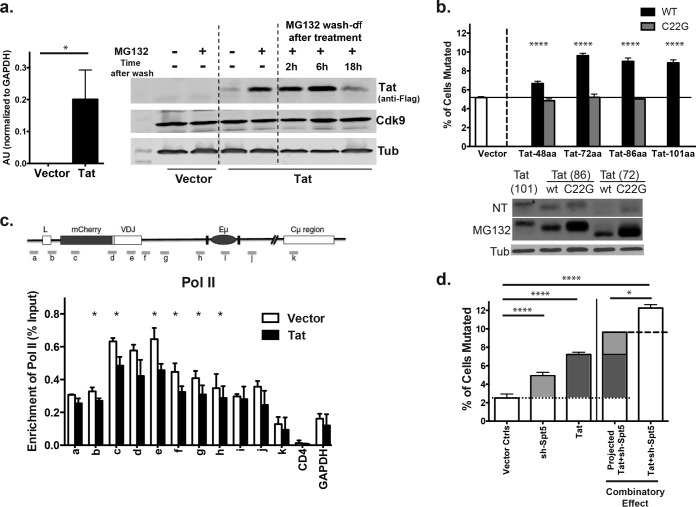
An extremely low-level HIV Tat protein is sufficient to promote SHM by modulating the Pol II transcriptional control machinery. (a) The mRNA level of the HIV-1 Tat gene was assessed by quantitative PCR in HIV-1 Tat-encoding or control vector-transduced Ramos B cells. AU, arbitrary units calculated from the ΔΔ*C*_*T*_ method normalized to GAPDH. The data shown are a representative result from 3 independent experiments, and the protein level of HIV-1 Tat-Flag tag fusion protein and the Cdk9 component of PTEF-B complex were assessed by Western blotting in Ramos cells transduced with control vector and not treated with MG-132 (lane 1) or treated with MG-132 for 4 h (lane 2). Ramos cells transduced with HIV-1 Tat-encoding vector untreated (lane 3) or treated with MG-132 for 4 h (lane 4). In lanes 5 to 7, MG-132 was washed out at the indicated time points after 4 h of treatment. Tubulin was blotted as a loading control, and the data shown here are a representative example from 3 independent transduction experiments. (b) Effect of indicated forms of HIV-1 Tat on SHM were assessed using Ramos SHM reporter cells induced to transport AID into the nucleus. C22G indicates the mutated forms of Tat that cannot bind P-TEFb. Expression levels of the truncated and mutant forms of HIV-1 Tat were assessed by Western blots of the various vector-transduced Ramos B cells. “NT” indicates samples that were not treated with MG-132. Western analysis results representative of 2 independent experiments are shown. Mutation analysis data represent one of four independent transduction and induction experiments and are shown as an average of duplicates or triplicates, with error bars indicating the standard deviation between the replicates. (c) Anti-Pol II ChIP assays were performed in Ramos reporter cells with vector control or Tat1 expression. Data were normalized to input after subtraction of IgG background and represent the average from three independent experiments. The CD4 gene, which is not transcribed in Ramos, was used as a negative control for Pol II occupancy, while GAPDH was a positive control. (d) The individual and synergistic effects of HIV-1 Tat and shRNA knockdown of the Spt5 DSIF complex component in enhancing SHM was assessed in the reporter Ramos cell line. Under the vector control condition, reporter cells were transduced with both empty vector and a scrambled shRNA construct. Mutation was determined by the loss of mCherry fluorescence. The predicted value of the simple additive effect through the combination of shRNA-Spt5 and Tat was calculated by adding the increase of SHM of the individual factors numerically. The data shown here are the representative result from 3 independent experiments and are shown as an average of duplicates or triplicates with error bars indicating the standard deviation between the replicates.

To explore the molecular mechanism of Tat-mediated enhancement of SHM, we first investigated the cellular level of Tat in the Tat-transduced human B cells. We found that although there was a substantial level of the steady-state Tat-1 mRNA (Fig. 2a, left), the steady-state level of Tat-1 protein was barely detectable (Fig. 2a right, lane 3 versus lanes 1 and 2). This suggests that (i) HIV Tat is tightly controlled at a posttranscriptional level in the Ramos human B cells and (ii) an extremely small amount of cellular Tat protein is sufficient to enhance the SHM process. The proteasome inhibitor MG-132 was able to dramatically increase the steady-state level of Tat-1 protein (Fig. 2a, right, lane 4) indicating a tight control of Tat protein stability through the proteasome-dependent degradation pathway in these B cells. Neither the level of Tat protein itself nor MG-132 affects the level of Cdk9—a major component of the P-TEFb complex (composed of Cdk9 and cyclin T1) with which Tat interacts in the cell (15) (Fig. 2a). Similar proteasome-mediated control also applies to the Tat from HIV-2 virus (see Fig. S2a in the supplemental material). We were unable to evaluate the effect of the increased levels of Tat protein associated with MG-132 treatment on V region mutation because the MG-132-treated cells did not survive long enough for AID-induced mutations to accumulate. This low level of Tat-1 had no significant effect on the steady-state mRNA levels of *AID* or the *Igh-V* region (Fig. S2b and S2c), so that is not the explanation of the increase in SHM (16, 17). We searched for other factors whose expression might have been influenced by Tat using microarray analysis (Fig. S2d), but only 6 of more than a million probes (see Table S1 in the supplemental material) revealed a >2-fold difference in expression that was statistically significant (*P* < 0.05; false-discovery rate [FDR], >0.5), and none of the genes represented by those 6 probes have known effects on SHM in B cells.

10.1128/mBio.02315-17.2 FIG S2  (a) The protein level Tat-2 of HIV-2 was determined using anti-Flag antibody. The mRNA level of *Igh-V* (b) and of the AID gene (c) was assessed by quantitative PCR and normalized to GAPDH in control or HIV-1 Tat-encoding vector-transduced Ramos B cells. Data are shown as a representative result from 3 independent experiments. (d) The gene expression microarray was conducted on reporter Ramos cells transduced either with the HIV Tat-encoding vector or with the control empty vector in 3 independent transductions. The volcano plot illustrates the difference in expression levels between the two conditions and its corresponding *P* value. Probes with significant expression differences are highlighted in blue (upregulation by Tat) or in red (downregulation by Tat). (e) Diagram of the functional subdomains of HIV-1 Tat and the mutated forms that were investigated in Fig. 2b. Download FIG S2, PDF file, 1.1 MB. Copyright © 2018 Wang et al. 2018 Wang et al. This content is distributed under the terms of the Creative Commons Attribution 4.0 International license.

10.1128/mBio.02315-17.3 TABLE S1  A gene expression microarray was conducted on reporter Ramos cells transduced with either with the HIV Tat-encoding vector or with the control empty vector in 3 independent transductions as described in the legend to Fig. S2d. Individual probes with significant differences in expression between the two conditions are listed. Download TABLE S1, PDF file, 0.1 MB. Copyright © 2018 Wang et al. 2018 Wang et al. This content is distributed under the terms of the Creative Commons Attribution 4.0 International license.

The HIV Tat protein is composed of multiple domains that are well characterized for their functions (15, 18, 19). To understand the molecular mechanism by which Tat enhances SHM, we first tested the effect of 3 different truncated forms of Tat-1: the minimal transactivation domain (1 to 48 aa), the first exon (1 to 72 aa), and the “short form” of Tat (1 to 86 aa) (Fig. S2e). We found that, albeit to a lesser extent, the minimal Tat transactivation domain (aa 1 to 48) was sufficient to enhance SHM (Fig. 2b). The region containing the nuclear localization signal of Tat (aa 48 to 72) was clearly required for the optimal enhancing effect, and the second exon of Tat (aa 72 to 101) seemed to be dispensable (Fig. 2b). Since Tat interacts with P-TEFb and the Pol II complex through the minimal transactivation domain, we concluded that the capacity of Tat to interact with the transcription machinery was essential for its enhancing effect on SHM. Consistent with this notion, a single amino acid mutation at cysteine 22 of Tat (C22G) that abolishes its interaction with P-TEFb (6, 20) also eradicated the capacity of all of the forms of the Tat molecule to enhance SHM (Fig. 2b). The loss of the SHM-enhancing capacity of the C22G Tat is not due to instability of the mutants because the protein levels of Tat(C22G) were in fact higher both at the steady state and after MG-132 treatment for at least two of the three truncated forms of Tat (72 aa and 86 aa) (Fig. 2b, bottom). Thus, Tat must interact with the P-TEFb complex to promote AID-mediated SHM.

We recently reported that single-stranded DNA (ssDNA) substrates of AID during SHM could come from premature transcription termination (9), the frequency of which relies on both Pol II processivity controlled by Spt5/Spt4 complex and the efficiency of releasing stalled Pol II complexes (21) by the P-TEFb complex. We hypothesized that HIV Tat decreases the efficiency of the recruitment of active P-TEFb complexes to the *Igh-V* loci, resulting in a less successful transition to elongation and more of a tendency for stalled Pol II to enter a degradation pathway (22). In line with our hypothesis, Pol II occupancy significantly decreased across the Ig variable region (Fig. 2c, sites c to f) in the presence of Tat but does not change at the transcriptional start site (Fig. 2c, site a), the intronic enhancer (Fig. 2c, sites i and j), the constant region (Fig. 2c, site k), the unexpressed CD4, or the highly expressed glyceraldehyde-3-phosphate dehydrogenase (GAPDH) control gene. Furthermore, our hypothesis predicts that Tat will act synergistically with the reduction of Spt5 level in enhancing the SHM process by further promoting premature transcription termination. In fact, while the expression of Tat or the reduction of cellular Spt5 alone resulted in a comparatively moderate increase in SHM frequency, the combination of these two events increased the SHM rate to a level that was significantly higher (Fig. 2d, lane 5) than their calculated additive effect (Fig. 2d, lane 4). Our data thus suggest that Tat could tip the balance toward early Pol II loss and premature transcription termination.

Overall, our data revealed an unexpected capability of HIV Tat to enhance AID-mediated SHM in the human B cells. The most stringent test of the sole effect of HIV Tat on B cells would require some sort of *in vivo* experiment, but this is not possible since interaction between Tat and P-TEFb is not conserved in nonprimates and Tat protein itself also changes infected T cells. Nevertheless, we believe that our findings using a human B cell line have physiological relevance for the following reasons. (i) Germinal center B cells closely interact with follicular helper T cells (Tfh) that host actively Tat-producing replicating HIV viruses throughout the infection even after the HIV titer is controlled by either the host immune system or ART (23). (ii) Based on the studies with Ramos cells reported here, paracrine Tat protein from infected T cells *in vivo* should be sufficient to provide the small amounts of intracellular Tat needed to promote SHM in bystander B cells and enhance genomewide genomic instability. (iii) Human immunoglobulin genes undergo an enormous amount of mutation, insertion, and deletion in HIV-infected individuals. This makes it quite plausible that HIV Tat proteins secreted from infected follicular T helper cells shape B cell physiology in the germinal centers, providing a missing link between HIV infection and its contribution to the unusually high frequency of mutations in the HIV broadly neutralizing antibodies that arise from multiple rounds of germinal center mutation and selection (24) and contributing to the persistent high risk of lymphomagenesis in AIDS patients in the post-ART era.

### Methods. (i) Cell lines and antibodies.

The wild-type human Burkitt’s lymphoma Ramos cell line and its derivative reporter Ramos cell line have been described previously (9). Briefly, the subclone of Ramos used in the reversion assay harbors an early stop codon in the *Igh*-*V* region that leads to the loss of surface IgM in those cells. The endogenous level of AID mediates constitutive SHM that reverts the early stop codon to a coding sequence resulting in the reappearance of surface IgM on those cells (16). The frequency of revertant cells from at least 24 single-cell clones was used to estimate the mutation rate by maximum likelihood in the cells for each experimental condition (25). The reporter Ramos cell line was established by first replacing the endogenous *Igh*-*V* region with an mCherry-*Igh*-*V* fusion fragment using recombinase-mediated cassette exchange (RMCE) and then transfecting the AID-ER fusion protein into those cells. Subclones to be studied were selected based on their capacity to undergo SHM in a 4-OHT (Sigma-Aldrich)-inducible manner. SHM was quantified by determining the frequency of cells that had a loss of mCherry fluorescence based on flow cytometry analysis.

The antibodies used in this study were anti-Flag (1:1,000 dilution [Rockland]), anti-CDK9 (1:1,000 dilution [Santa Cruz]), and antitubulin (1:2,500 dilution [Sigma-Aldrich]). MG-132 was purchased from EMD Millipore and was used to treat the indicated cells at a concentration of 5 µM for 4 h before analysis. The HIV-1 Tat 101 construct was kindly provided by Joan Berman. The HIV-2 Tat and wild-type HIV-1 Tat 86 form and the HIV-1 Tat 86(C22G) mutant construct were provided by the NIH AIDS Reagent program.

### (ii) Lentiviral transduction of Tat expression and shRNA.

All the short hairpin RNA (shRNA) constructs were obtained from the human TRC library (Thermo Scientific) with sequences listed previously (9). Control shRNA (Ctrl-shRNA) is the SHC002 construct from Sigma-Aldrich. Lentiviral particles containing the designated shRNA or the exogenous expression vector of Tat-Flag protein and its mutant forms were prepared by the shRNA Core facility at Albert Einstein College of Medicine. Ramos cells were transduced with an ~3:1 multiplicity of infection (MOI) and were subjected to puromycin (Gibco, Life Technology) selection for 7 to 9 days in the case of shRNA. Successful knockdown of targeted genes was verified by real-time PCR and Western analysis as confirmed previously (9). Ramos cells expressing the Tat gene or its derivative forms were sorted based on the coexpression of the GFP marker that is on the same lentiviral construct under control of an independent promoter.

### (iii) Mutation analysis.

To obtain the mutation pattern, the cells that had lost their mCherry fluorescence were sorted by flow cytometry and their genomic DNA was extracted (Qiagen). The mCherry-*Igh-V* fusion region was amplified using Pfu Turbo (Agilent), cloned into the sequencing vector, and Sanger sequenced in both directions to cover the whole ~1.3-kb region. Sequencing data were then aligned by ClustalW2 and analyzed using SHMTool (http://shmtool.montefiore.org).

### (iv) Chromatin immunoprecipitation assay.

Ramos cells were fixed in 1% formaldehyde for 10 min and then quenched with 125 mM glycine for 5 min. Fixed chromatin was harvested from cells by SDS lysis buffer with a protease inhibitor and sheared by sonication to an average length of 200 to 500 bp. After preclearance with protein G Dynabeads (Thermo Fisher Scientific catalog no. 10004D) for 2 h by rotation at 4°C, immunoprecipitation was performed with either specific antibodies or normal IgG as a negative control and incubation overnight at 4°C. This was followed by pulldown assays with protein G Dynabeads. DNA in the chromatin-protein complex was then extracted using Chelex-100 resin (Bio-Rad catalog no. 1421253) and quantified by real-time PCR.

### (v) Gene expression.

The gene expression level of individual genes of interest was assessed by real-time PCR using the threshold cycle (ΔΔ*C*_*T*_) method with SYBR green PCR master mix (Life Technology or KAPA Biosystems). In the case of the genomewide transcription study, RNA was purified from the indicated cell type and assayed on the Affymetrix Hugene 2.0 ST array according to the manufacturer’s instructions and was performed by the Genomics Core at Albert Einstein College of Medicine.

### (vi) Statistical analysis.

All statistical analyses were conducted using Prism 6 software. Error bars represent standard deviations (SD) among independent experiments or variation among replicates, as indicated in the figure legends. In cases in which multiple experiments were compiled, a paired Student’s *t* test was used. Throughout the article, * indicates *P* < 0.05, ** indicates *P* < 0.01, *** indicates *P* < 0.001, and **** indicates *P* < 0.0001.
