# Supplementary figures and images for: Human Immunodeficiency Virus Tat Protein Aids V Region Somatic Hypermutation in Human B Cells
Source: mBio. 2018 Apr 17;9(2):e02315-17. doi: 10.1128/mBio.02315-17 (PMC5904410; doi:10.1128/mBio.02315-17)

## Supplemental figure 2:

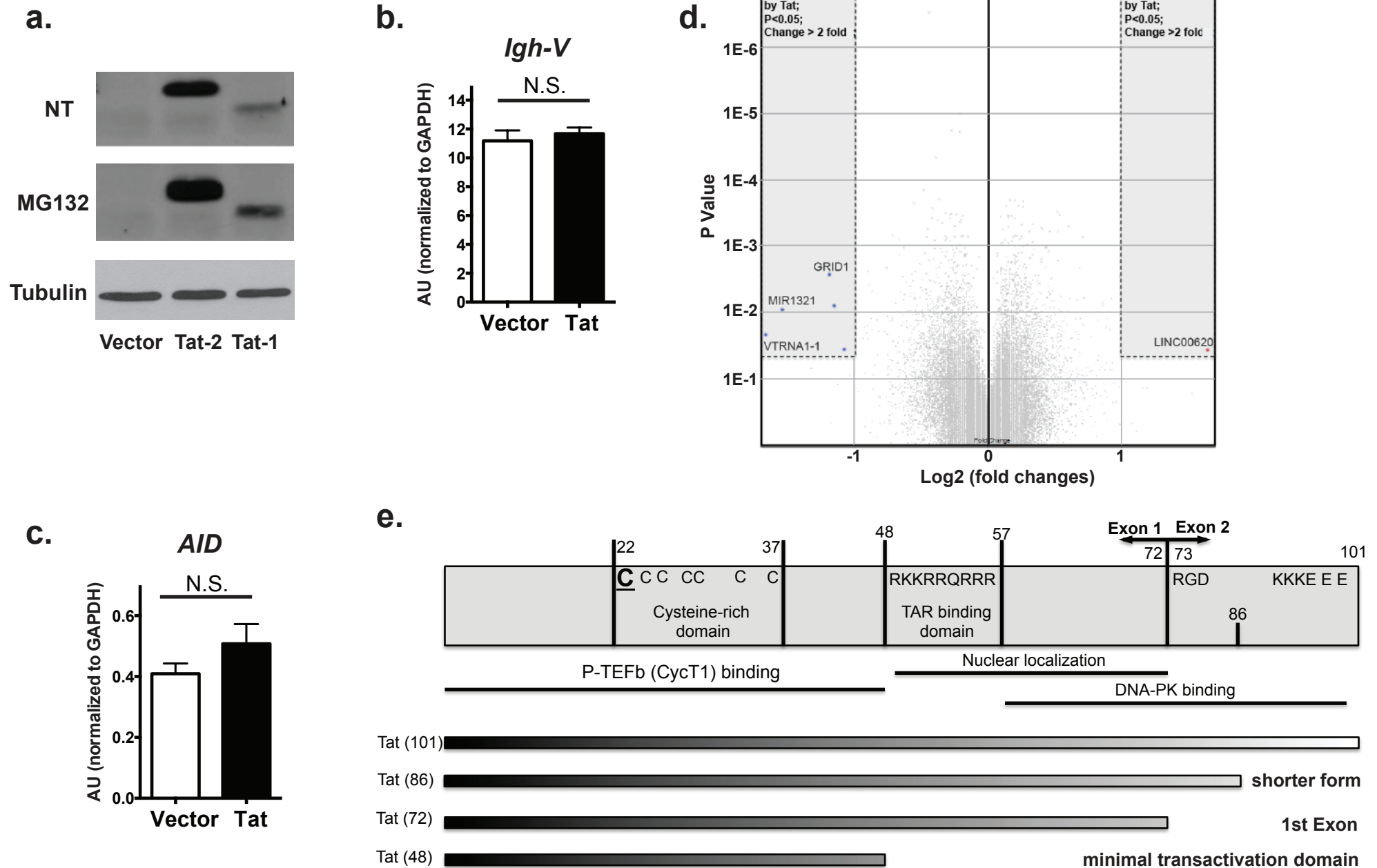

Supplement: FIG S2 [file mbo001183822sf2.pdf]
