# Supplementary material for: Human Immunodeficiency Virus Tat Protein Aids V Region Somatic Hypermutation in Human B Cells
Source: mBio. 2018 Apr 17;9(2):e02315-17. doi: 10.1128/mBio.02315-17 (PMC5904410; doi:10.1128/mBio.02315-17)
Supplement: TABLE S1 [file mbo001183822st1.pdf]

Supplemental Table 1:

Transcript Cluster ID	Fold Change	ANOVA p-value	FDR p-value	Gene Symbol	Chromosome	Start	Stop
16950987	3.14	0.037616	0.718794	LINC00620	chr3	13779558	13779629
16729710	-2.12	0.035989	0.718794	NA	chr11	85864269	85864376
17125996	-2.24	0.007807	0.674022	NA	chrU	1	54
16716179	-2.29	0.002765	0.674022	GRID1	chr10	88062583	88062678
17105179	-2.91	0.009294	0.674022	MIR1321	chrX	85090785	85090863
16990199	-3.19	0.021966	0.704987	VTRNA1-1	chr5	140090860	140090958
